# Effects of SNPs and alternative splicing within *HGF* gene on its expression patterns in Qinchuan cattle

**DOI:** 10.1186/s40104-015-0059-3

**Published:** 2015-12-22

**Authors:** Hanfang Cai, Yang Zhou, Wenchao Jia, Bowen Zhang, Xianyong Lan, Chuzhao Lei, Xintang Fang, Hong Chen

**Affiliations:** College of Animal Science and Technology, Shaanxi Key Laboratory of Molecular Biology for Agriculture, Northwest A&F University, Yangling, Shaanxi 712100 People’s Republic of China; Institute of Cellular and Molecular Biology, Xuzhou Normal University, Xuzhou, Jiangsu 221116 China

**Keywords:** Alternative splicing, Expression, HGF, Qinchuan cattle, SNPs

## Abstract

**Background:**

Identification of genetic variants, including SNPs (Single Nucleotide Polymorphisms), CNVs (Copy Number Variations) and alternative splicing, within functional genes has received increasing attention in animal science research. HGF (Hepatocyte Growth Factor) is a very important growth factor that works as a mitogen or a morphogen during tissue growth, development and regeneration. However, to date, the functions of genetic variants within the bovine *HGF* gene, particularly their effects on mRNA expression, have not been determined well.

**Results:**

The present study aimed to perform association analysis between genetic variants and mRNA expression for the bovine *HGF* gene in Qinchuan cattle using various strategies, including PCR-RFLP (Restriction Fragment Length Polymorphism), qPCR (Quantitative Real-time quantitative PCR), TA cloning, DNA sequencing and bioinformatics analysis. A total of five SNPs were identified and only SV1 locus significantly affected *HGF* mRNA expression in fetal skeletal muscle (*P* < 0.05). Heterozygous genotype individuals showed significantly higher *HGF* expression (*P* < 0.05), which was significantly greater in the “CTCCAGGGTT” combined genotype than that in the “CCCCGGGGTT” combined genotype (*P* < 0.05). In addition, two alternative splicing variations, HGF-W and HGF-M, were identified, which resulted from alternative 3′ splice sites of exon 5, and HGF-W showed higher mRNA levels than HGF-M in all tissues.

**Conclusion:**

In summary, genetic variations within the *HGF* gene affected mRNA expression. These findings provide new insight into the molecular characteristics and functions of bovine HGF.

## Background

HGF, firstly described as a potent mitogen for hepatocytes, has mitogenic [1], motogenic, morphogenic [2], anti-apoptotic [3] and anti-fibrotic [4] activities in various cell types, via c-Met, the specific receptor of HGF [5]. Three signaling pathways, including PI3K (phosphatidy linositol 3-kinase), STAT (signal transducer and activator of transcription) and MAPK (mitogen activated protein kinase) [6–8], regulating growth and development in mammals and are related to the HGF-Met pathway. However, the functions of the bovine HGF remain unclear.

SNPs are commonly used as molecular markers. Exonic SNPs have a direct effects on the properties of proteins, while SNPs within introns and untranslated region can affect the expression and splicing of mRNA [9, 10]. Numerous studies have shown that SNPs in the *HGF* gene are associated with normal growth and development [11–13]. However, it remains unclear that how these mutations affect gene expression, including the bovine *HGF* gene examined in this study.

Alternative splicing, sequences of pre-mRNA could be alternatively included into the mature mRNA or removed, has been widely examined in genomes studies. Increasing evidences have demonstrated that alternative splicing contributes to protein localization, enzymatic properties, and protein binding domains [14–16], and so on. Three transcript variants, X1 (XM_005205314), *X*2 (XM_005205315) and X3 (XM_005205316), were predicted to be present in Hereford cattle. Moreover, biologically active rat HGF protein contains 697 or 692 amino acids [17], indicating that there are two alternative splicing transcripts of HGF in mice. However, there have been no reports regarding HGF splicing variations in Chinese cattle.

In this study, firstly, the effects of SNPs in the bovine *HGF* gene on mRNA expression were conformed and found that variants within bovine *HGF* gene were associated to its mRNA levels. Secondly, splicing variants and their tissue expression were investigated. These results provide a foundation for further studies on the bovine HGF.

## Methods

### Sample collection and genomic DNA/total RNA isolation

Skeletal muscle samples from 48 male and 37 female fetal Qinchuan cattle were obtained for genomic DNA and total RNA isolation to detect the correlation between mutations and relative mRNA expression levels. Different tissue samples, including skeletal muscle, liver, heart, kidney, lung and spleen from fetal (5 months of gestation) and adult (24 months old) of Qinchuan cattle (*n* = 5, respectively) were collected for total RNA isolation to evaluate tissue expression.

Total RNA was extracted from different tissues using the Trizol kit (Takara, Japan). cDNA was synthesized according to the PrimeScript RT Reagent Kit (Perfect Real Time) (Takara, Japan). Genomic DNA from skeletal muscle was extracted by proteinase K digestion, chloroform extraction and absolute ethanol precipitation.

### Genotyping

In order to evaluate the effect of SNPs in the *HGF* gene on its mRNA expression, five mutations were identified. Four known mutations (SV1: intron 1, AC_000161.1:g.288 T > C; SV3: exon 13, AC_000161.1:g.72801G > A; SV4: intron 17, AC_000161.1:g.77172G > T and SV5: intron 18, AC_000161.1:g.77408 T > G) were detected as described by Cai et al. [13]. In the previous resequencing analysis of Qinchuan cattle [unpublished observations, Xu et al.], SV1, SV3 and three novel mutations were detected in the *HGF* gene. However, only one novel mutation (SV2: AC_000161.1:g.47944C > G) in exon 8 was identified as a novel SNP locus in the tested individuals in this study. Primer P-BglII (Table 1) was designed in PCR-RFLP to identify individual genotypes of the SV2 locus. The PCR product was digested with *Bgl*II and detected using 3 % agarose gel electrophoresis.

**Table 1:** Primers used for mutation detecting, cloning and expression survey

Primer	Sequences (5′–3′)	Size, bp	Tm, °C
P-BglII	F: TTTACCAATAGCCCACAG	229	53
	R: CATTCTGCCTACTGAAATG		
P-HGF-CDS	F: TCTGAGTCGGAAGAGGGT	2, 250	54
	R: ATAAGGCACCACAGTTGTAG		
P-Q-HGF	F: ACCAATGTGCCAATAGATG	229	60
	R: TTAGTGATAGATACCGTCCC		
P-qHGF-W	F: GTATCATTGGTAAAGGCGGTAG	119	60
	R: ATAGCTCGAAGGCAAAAAG		
P-qHGF-M	F: GTATCATTGGTAAAGGCGGTAG	109	60
	R: CCCCGATAGCTGTGTTCG		
P-Q-GAPDH	F: TGTTTGTGATGGGCGTGAACCA	154	60
	R: ATGGCGTGGACAGTGGTCATAA		
P-HGF-AS	F: TTGGTAAAGGCGGTAGCT	205/190	58
	R: TAGCGTACCTCTGGATTGCT		

### Identification of bovine HGF splicing variants and bioinformatics analysis

Sixty cDNA samples were collected from 6 tissues of 10 cattle individuals (5 fetal and 5 adults) to prepare a cDNA pool, which was used as a template for amplification using the primer P-HGF-CDS (Table 1), which covers the whole coding region of bovine *HGF* gene based on the reference sequence (NM_001031751.1). The product was purified and ligated into the pGEM-T easy vector (Promega, USA) and surveyed by sequencing. Sequences were compared with the reference sequence by BLAST (http://blast.ncbi.nlm.nih.gov/Blast.cgi). SMART (http://smart.embl-heidelberg.de) was used to predict the protein domains of bovine HGF.

### Quantitative Real-time quantitative PCR (qPCR)

qPCR was performed using gene specific primers designed by Primer 5.0, with glyceraldehyde-3-phosphate dehydrogenase (GAPDH) as an endogenous control (Table 1). The PCR specificity of these primers was evaluated using Primer-Blast (http://www.ncbi.nlm.nih.gov/tools/primer-blast/index.cgi?LINK_LOC=BlastHome). The reaction was carried out with SYBR premix ExTaq II (Takara, Japan) and a CFX 96^™^ Real Time Detection System (Bio-Rad, USA) according to the manufacturer’s instruction. The qPCR was performed in 20 μL of reaction mixtures consisting of 50 ng genomic DNA, 10 μL SYBR Premix ExTaq II (Takara, Japan), and 20 pmol each primer. Thermal cycling conditions were: 95 °C for 1 min, followed by 40 cycles of 95 °C for 10 s, 60 °C for 30 s. Melting curve analysis was performed to confirm specific amplification. Each PCR was performed in triplicate.

### Statistical analysis

The relative expression ratios were calculated using the following formula as described by Schmittgen and Livak the 2^-ΔΔCt^ method [18], in which ΔΔCt = ΔCt_(test)_ ‐ ΔCt_(Calibrator)_, ΔCt_(test)_ = Ct_(target gene)_ ‐ Ct_(reference gene)_, ΔCt_(Calibrator)_ = Ct_(target Calibrator)_ ‐ Ct_(reference Calibrator)_. The data were expressed as the mean ± SE (Standard Error).

After genotyping, SPSS 18.0 software was applied to analyze the association betwween genotypes and genotypic combinations with expression levels in Qinchuan cattle using one-way analysis of variance. The following adjusted linear model as follow was applied: Y_il_ = *μ* + G_i_ + S_l_ + G_i_S_l_ + e_il_, where Y_il_ is the expression; *μ* is the overall mean; G_i_ is the effect of genotypes when this model was used to analyze the association between a single locus and mRNA expression level, and G_i_ is the effect of genotypic combinations when this model was used for the association analysis between combined genotypes and expression level; S_l_ is the effect of sex; and e_il_ is the residual effect. The differences in expression levels between two alternative splicings in every tissue was analyzed using Student *t* test. The threshold of significance was *P* < 0.05.

## Results

### HGF mRNA expression based on genotypes in skeletal muscle of fetal bovine

**Fig. 1:**
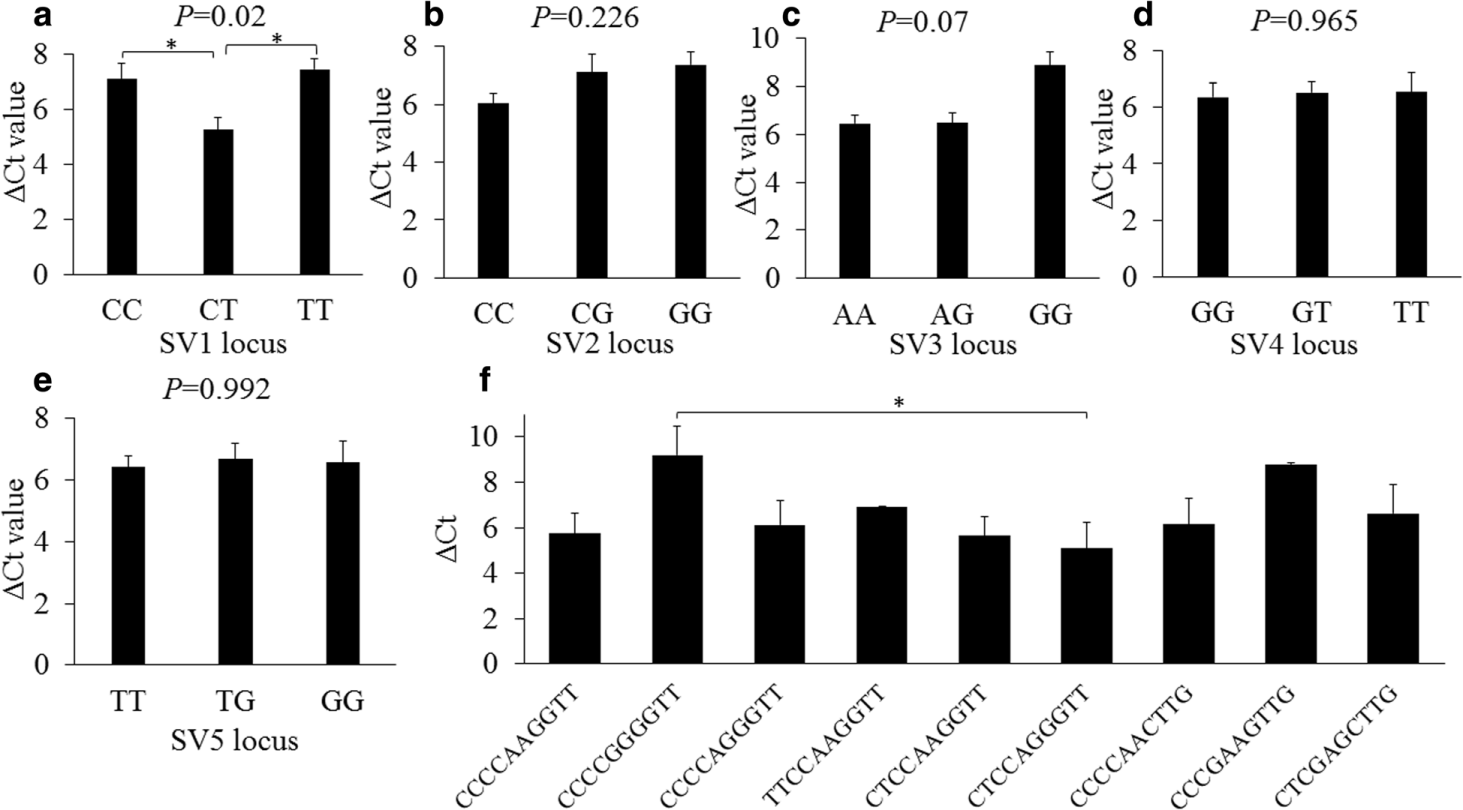
Association between bovine *HGF* gene expression in fetal skeletal muscle and its genetic variants. **a**–**e** The association between bovine *HGF* gene expression level and the 5 SNPs loci. Only SV1 locus has a significant effect on HGF expression (*P* < 0.05). **f** The comparison of HGF expression among different genotypic combinations. The bars represent the mean of ΔCt value ± SE. “*” indicates the significant difference in the HGF expression (*P* < 0.05)

Firstly, the expression of *HGF* gene (primers P-Q-HGF was used, Table 1) in female cattle was compared with that in male cattle using the *t* test, but no significant difference (*P* > 0.05) was observed. To evaluate the effect of mutations on HGF mRNA expression, the relationships between five SNPs locus and HGF expression in the skeletal muscle of fetal cattle were analyzed (Fig. 1a–e). SV1 (57 cattle with CC genotype, 6 cattle with TT genotype and 22 cattle with CT genotype) was significantly associated with relative *HGF* gene expression levels (*P* < 0.05) (Fig. 1a). Particularly, individuals with the CT genotype had notably higher expression than those with others genotypes (*P* < 0.05). However, there was no significant differences between the HGF mRNA expression levels of fetal skeletal muscle with other loci (*P* > 0.05). In addition, the effects of multiple SNPs on expression were evaluated based on the association of genotypic combinations and HGF mRNA levels. Diplotypes with individual numbers less than 2 did not contribute to the association analysis (Fig. 1f). As a result, the “CCCCGGGGTT” diplotype with the highest HGF expression and the “CTCCAGGGGTT” diplotype with the lowest expression showed significant difference (*P* < 0.05). The general linear model analysis revealed there was no significance relationship between the interaction of sex and genetic mutations and expression (*P* > 0.05). Finally, genetic variations within the bovine *HGF* gene were significantly associated with its mRNA expression.

### Identification and verification of alternative splicing in bovine HGF

**Fig. 2:**
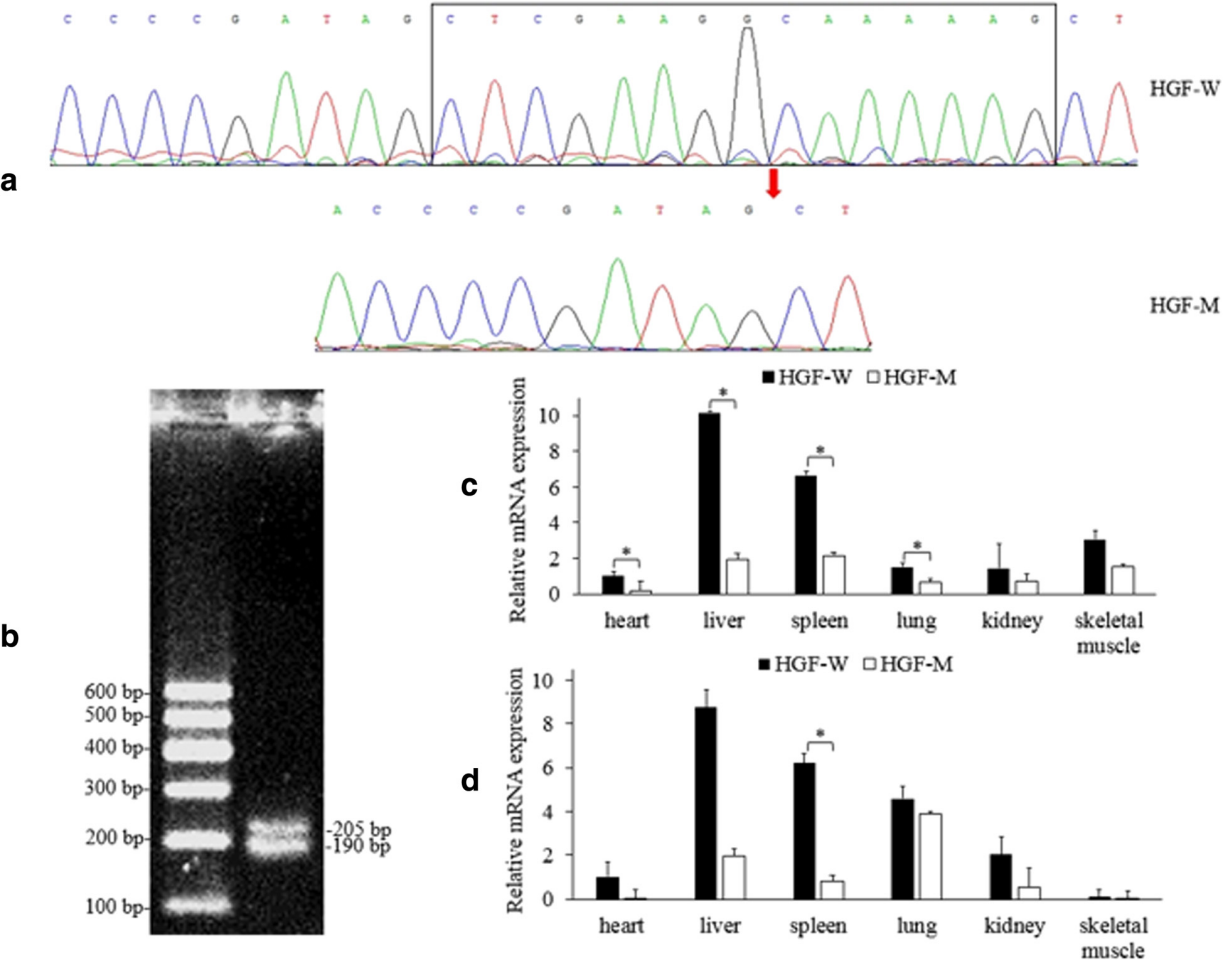
Comparison of HGF-W and HGF-M. **a** The sequence differences between HGF-W and HGF-M. These are the reversed sequencing maps. **b** The detecting of HGF-W and HGF-M by agarose gel electrophoresis. DNA molecular weight marker is DNA MarkerI. Tissue expression of HGF-W and HGF-M in fetal **c** and adult **d** cattle were shown. Gene expression was normalized against HGF-W expression in heart. Each column denotes the mean ± SE. “*” indicates a significant difference at *P* < 0.05

**Fig. 3:**
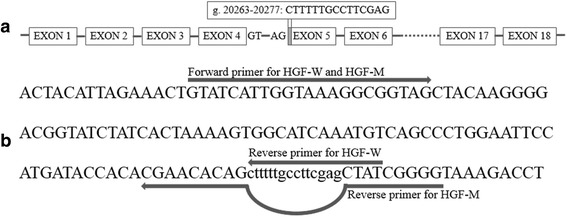
Alternative splicing of bovine *HGF* gene. **a** The genomic location of splicing fragment (*gray region*) in HGF-M was compared to HGF-W. Boxes show the exons and lines represents introns. Dashed line indicating the partial genomic structures are not listed. **b** Positions of primers for detecting the HGF-W and HGF-M mRNA expression level. Lower cases show the splicing fragment sequence. The bow line indicates that the reverse primer for HGF-M skips the splicing fragment

Based on the sequencing results of the pGEM-T-HGF plasmids, two transcript variants of bovine HGF were identified, including HGF-W and HGF-M (Fig. 2a). HGF-W, encoded for 730 amino acids and was 15 bp longer than HGF-M because of the presence of alternative 3′ splice sites of exon 5 (Fig. 3a). Sequence alignment analysis showed that GT-AG was the 5′ splice donor and 3′ splice acceptor site in all exon-intron boundaries in the genomic sequence of the bovine *HGF* gene, while AG was the 3′ end of spliced site. In order to verify whether such alternative splicing occurred, a pair of primers (P-HGF-AS) covering the splicing fragment were designed (Table 1). The PCR product which used a cDNA pool as template was detected using 3 % agarose gel electrophoresis (Fig. 2b). Two clearly visible bands with a differential of 15 bp were observed, indicating that alternative splicing had occurred.

### Bioinformatics analysis

SMART analysis showed no differences in protein domain types between HGF-W and HGF-M containing an N-terminal transmembrane segment, followed successively by a PAN_AP domain, four tandem Kringle domains, and a C- terminal Tryp_SPc domain, which was similar to the reported HGF structures in other species [17]. The bovine HGF-W sequence was used as a query sequence to search for similar sequences using BLAST. The amino acid sequence of Qinchuan cattle HGF shared 99 % identity with all tested species, indicating that HGF protein was highly conserved during evolution. Furthermore, splicing events are also observed in human (NP_001010932.1 and NP_000592.3), dog (XP_005630939.1 and NP_001002964.1) and other species.

### Quantification of alternative splicing in bovine HGF

To evaluate the tissue expression of HGF-W and HGF-M, two pairs of specific primers (P-qHGF-W and P-qHGF-M) were designed (Fig. 3b and Table 1). As shown in Fig. 2c–d, these two splicing variants were measured in six tested tissues of fetal and adult cattle. Interestingly, the expression of HGF-W was higher than that of HGF-M in all tested tissues, suggesting that HGF-W was the domain alternative splicing variant. In particular, the statistical differences were significant in the heart (*P* = 0.046), liver (*P* = 0.006), spleen (*P* = 0.025), lung (*P* = 0.030) in fetal bovine. However, in adult bovine, a statistical difference was observed only in the spleen (*P* = 0.015). The expression level of HGF-W in the liver was the highest among the expression levels of tissues in both fetal and adult cattle. HGF-M expression was the highest in the spleen of fetal cattle, but in the lung of adult cattle. In contrast, the lowest expression was observed in the heart in both stages. These two alternative splicing events within the bovine *HGF* gene resulted in varying expression levels among different tissues.

## Discussion

Bioinformatics analysis showed that HGF was highly conserved across species, and thus it was hypothesized that this protein plays a vital role in the growth and development of Qinchuan cattle. Given the emerging roles of HGF in normal growth and development, the identification of genetic variations, transcript variations and expression of HGF appears an essential step for further studies on HGF in bovine growth and development.

A large number of studies have suggested that gene polymorphisms are associated with expression, which was also confirmed in this study. Five SNPs in the bovine *HGF* gene were identified, only one SNP in intron 1 was significantly associated with HGF expression in Qinchuan cattle. In some genes, intron 1 is in the range of promoter and cis-regulating elements, which may play an essential role in controlling transcription and expression [19]. Thus, SV1 may affect HGF mRNA expression.

It is well-known that all muscle fibers, the structural units of skeletal muscle, are formed in the prenatal stage [20]. During this period, HGF helps myogenic precursor cells to move from somite to limb buds. Postnatal muscle growth invovles muscle fiber hypertrophy, hyperplasia [21] and regeneration [22]. During regeneration, satellite cells may be activated, resulting in the expression of HGF and its receptor [23]. Skeletal muscle is a major part of the animal body and is related to animal health, growth traits and animal products [24]. Thus, the fetal skeletal muscle was used to estimate the impact of genotypes on mRNA expression. The result showed that SV1 was significantly related to the expression level of the *HGF* gene. Additionally, SV1 was significantly associated with growth traits in Chinese cattle [13]. These resluts suggested that SV1 affects HGF mRNA expression in fetal skeletal muscle, and then influences the function of bovine HGF and growth traits. These findings provide insight into the molecular mechanism of HGF post-transcriptional regulation.

Alternative splicing is predicted to exist in more than 90 % human genes [25]. Since alternative splicing is a primary source of biological complexity in mammals [26], two transcript variants and their expression of HGF was identified. Various mechanisms are involved in alternative splicing: alternative 5′ splice selection, alternative 3′ splicing selection, exon skipping and intron retention [27]. Sequence alignment indicated that HGF-M is subject to exon skipping and results from the alternative 3′ splicing site selection of exon 5. According to previous studies, two main mechanisms create this 3′ splice site: random mutations in intronic sequences and RNA editing, which can lead to spliceosome recognition and exonization [27]. However, no other mutations were found in the splicing region according to the DNA pool sequencing result [13] and resequencing analysis [unpublished observations, Xu et al.]. In contrast, AG was located in the 3′ end of splicing region (Fig. 3), which was the same as the 3′ splice acceptor site in exon-intron boundaries of the bovine *HGF* gene. Therefore, this 3′ splice site in bovine *HGF* may have been induced by RNA editing.

qPCR showed that two splicing variants were ubiquitously expressed and that the expression of HGF-W was higher than HGF-M in all examined tissues. According to an NCBI search, the two alternative splicing variations exist in many species, including NP_001010932.1 and NP_000592.3 in humans. In SMART analysis, the deduced protein domains of HGF-W were compared to that of HGF-M. The result showed that the splicing fragment was located in 4 Kringle domains, which were connected via the HGF receptor, Met [28]. Thus, alternative splicing of HGF-M may influence the HGF-Met pathway by changing the combination of HGF to Met. This hypothesis is consistent with the results of previous studies examining alternative splicing altering the binding to other proteins.

## Conclusions

In summary, genetic variations within the bovine *HGF* gene significantly affect gene expression and then works on gene final function. This is the first report describing two bovine HGF transcript variants in Qinchuan cattle, which were confirmed without tissue specificity and species specificity. These results increasing the understanding of molecular mechanisms of bovine HGF.

## Abbreviations

ANOVA: one-way analysis of variance
CNVs: Copy number variations
HGF: Hepatocyte Growth Factor
MAPK: mitogen activated protein kinase
PI3K: phosphatidy linositol 3-kinase
qPCR: Quantitative Real-time quantitative PCR
SNPs: Single Nucleotide Polymorphisms
STAT: signal transducer and activator of transcription
UTR: untranslated region
